# Analysis of clinical audiological characteristics in children with Williams syndrome in China

**DOI:** 10.1186/s13023-025-03650-2

**Published:** 2025-05-20

**Authors:** Fangfang Li, Bin Xu, Jiyang Shen, Weijun Chen, Junxia Guo, Dan Yao, Jie Shao, Chai Ji

**Affiliations:** 1 Department of Child Health Care, Children’S Hospital, Zhejiang University School of Medicine, National Clinical Research Center for Child Health, Hangzhou, China; 2 Department of Otorhinolaryngology-Head and Neck Surgery, Children’S Hospital, Zhejiang University School of Medicine, National Clinical Research Center for Child Health, Hangzhou, China

**Keywords:** Williams syndrome, Audiology, Children

## Abstract

**Background:**

Williams Syndrome (WS) is a neurodevelopmental disorder caused by microdeletion on chromosome 7. Hearing loss (HL) is common in this population but is rarely taken seriously. Previous studies had small sample sizes and mixed conclusions, and few studies have investigated HL in children with WS.

**Objectives:**

To investigate audiological characteristics of children with WS, analyze the influence factors, and to provide scientific basis for further improvement of ear and hearing care in children with WS.

**Methods:**

Children with WS aged 0-18yrs, followed up in the Department of Pediatric Healthcare of the Children’s Hospital of Zhejiang University School of Medicine from June 2020 to June 2024 were enrolled in this study. Children aged 0-18yrs who came in the same period for health examination were matched as the control group. Both groups underwent a series of audiological examinations such as tympanogram, distortion product otoacoustic emission (DPOAE), auditory brainstem response (ABR) and pure-tone audiometry (PTA), to analyze the audiological characteristics of WS at different ages, and their difference with control group. Tympanogram and DPOAE were suggested to retest 1 year later and the results of first and second test were also compared.

**Results:**

Tympanogram and DPOAE were completed in 130 WS and control subjects, ranging in age from 1.0 to 12.4 years in the WS group and 0.8–13.1 years in the control group. The passing rate of tympanogram and DPOAE in WS was significantly decreased when compared with control group (*p* < *0.05*), and these differences were found in all age groups. The lower DPOAE passing rate still remain after the tympanogram abnormal data were excluded. The SNR of 2000–5000 Hz were statistically lower in children with WS after tympanogram, DPOAE abnormal data were excluded. Tympanogram and DPOAE were rested in 25 WS 1 year later, and no significant difference was found in the passing rate of these test. ABR tests were completed in 28 WS and 44 control subjects, ranging in age from 0.7 to 5.2 years in the WS group and 0.4–5.2 years in the control group. Threshold of ABR in WS was higher than control group. The latency of wave I, III and the interpeak latency I-III in WS were significantly longer (*p* < *0.05*), and the interpeak latency III-V was significantly shorter than that in control group (*p* < *0.05*). PTA were completed in 20 WS and 28 control subjects, ranging in age from 5.9 to 13.7 years in the WS group and 5.9–12.5 years in the control group. 50% of WS was assessed as HL by PTA, with conductive hearing loss (CHL) in 60%, sensorineural hearing loss (SNHL) in 20% and mixed hearing loss (MHL) in 20%, most were mildly. The threshold of 250–8000 Hz in WS group were significantly higher than that in control group (*p* < *0.05*), either in air or bone conduction.

**Conclusions:**

This study revealed that children with WS frequently exhibit middle and inner ear dysfunction, often accompanied by HL or subclinical cochlear impairment, which can emerge before age 3. Prolonged ABR latency indicates delayed auditory nerve myelination, while shortened interpeak latency III-V may serve as an electrophysiological marker in this population. Long-term, regular hearing follow-up is recommended to enable early HL detection and timely treatment of contributing conditions.

## Background

Williams Syndrome (WS) is a neurodevelopmental disorder resulting from the loss of 24 to 28 heterozygous protein-coding genes located on chromosome 7q11.23, with an incidence rate of 1 in 7,500 to 20,000 [1, 2]. The common clinical phenotypes of this syndrome include distinct facial features, cardiovascular disease, connective tissue abnormalities, growth retardation, intellectual disability, and are often associated with hearing loss (HL) [3–5]. Genotype–phenotype evidence is strongest for ELN, the gene encoding elastin, which is responsible for the vascular and connective tissue features of WS, and for the transcription factor genes GTF2I and GTF2IRD1, which are known to affect intellectual ability, social functioning and anxiety [1]. The remaining genes were found to have some correlation with phenotype, but the specific pathogenesis remains unclear.

Hearing problems in WS often receive insufficient attention when compared with other clinical phenotypes in China. Even if the hearing screening fails during the follow-up visit, parents often do not proceed with further diagnostic hearing tests, citing their perception that their child is sensitive to sound and the difficulty in obtaining cooperation from the child during testing. As an important part of human perception, hearing ability greatly affects children’s language and cognitive ability. Individuals with mild HL may miss 10% of speech signals when speakers are more than 3 feet away. This percentage may increase if background noise exists [6]. Research indicates that children with mild HL perform significantly worse in speech recognition, auditory temporal processing, cognitive tasks in noisy environments, and academic achievement compared to their peers with normal hearing [7–9]. Individuals with mild to moderate HL exhibit significantly higher rates of language delay, behavioral issues, and attention deficits. Studies indicate that children with mild to moderate HL perform worse in speech production, sentence recall, language expression, and word reading compared to typically developing peers [10]. Even monaural HL can affect children’s language ability [11]. Neglecting HL in children with WS significantly hinders their cognitive development and language acquisition. And these may also have a great impact on their quality of life [12].

Auditory hypersensitivity was found in 90% of WS [13]. Consequently, HL in this group often does not receive adequate attention from family members or clinicians outside the fields of audiology or otolaryngology. It has been reported that 84% of individuals with WS fail to pass the hearing test, but those around them do not pay timely attention [14]. Several studies have indicated that progressive HL is common in individuals with WS [15]. Furthermore, there may be subclinical hearing dysfunction prior to the onset of HL, however, the timing of these hearing problems remains unclear [16]. Despite the high incidence of HL in this group, the identified genes associated with HL do not currently overlap with the deletion genes found in WS [17, 18]. This raises the question of whether the HL in this population is really caused by gene deletion? Human auditory pathway includes outer ear, middle ear, inner ear, belong to the peripheral auditory system, and auditory nerve and auditory cortex, belong to the central auditory system. The peripheral auditory system is fully functional at birth while the central auditory system continues to mature after birth. Any abnormality of any part of the pathway will affect the brain’s auditory perception. WS is a group of etiologically homogenous populations, and about 95% share the same gene deletion [19]. It provides an opportunity to understand the deleted gene, its physiology, and the therapeutic issues related to HL by studying the hearing perception and processing ability of this population. Based on a larger sample of children with WS, this study attempted to compare the differences in hearing performance between this population and the control group, as well as the changes between different age groups through hearing test such as tympanogram, distortion product otoacoustic emission (DPOAE), auditory brainstem response (ABR) and pure-tone audiometry (PTA), to determine whether HL is typical in this population and the time of HL occurrence, hoping to provide clinical basis for hearing follow-up and intervention in the target population in the future.

## Material and methods

Children with WS who were followed up from June 2020 to June 2024 in the Department of Child Health Care of Children’s Hospital, Zhejiang University School of Medicine, were enrolled as study group. Children who came in the same period for health examination were matched as the control group. This study was approved by the Medical Ethics Committee of Child Health Care of Children’s Hospital (2019-IRB-122), and all subjects’ guardians signed informed consent.

### Inclusion and exclusion criteria for subjects

Inclusion criteria for study group: 1) Children with WS confirmed by genetic testing such as Fluorescence in situ hybridization (FISH), Multiplex ligation-dependent probe amplification (MLPA), Chromosomal microarray analysis (CMA) or Whole exome sequencing (WES). 2) Age range 0–18 yrs old. Inclusion criteria for control group:1) Children came for health examination. 2) Age range 0–18 yrs old.

The common exclusion criteria for both the study group and the control group: 1) Subjects who can’t cooperate with the test. 2) Subjects whose family members reject to participant. 3) Children with traumatic brain injury, epilepsy, other severe physical or mental illness that can cause brain dysfunction.

### Other exclusion criteria that occur during data analysis

Any ear that does not pass the tympanogram or DPOAE will be excluded when making the DPOAE SNR comparison. In addition to the above two items, the ears with ABR threshold greater than 30 dB nHL will also be excluded in the process of ABR latency analysis.

### Audiologic assessment

Before the test, a routine ear examination is performed by an ENT doctor and the external ear canal is cleaned. All hearing tests are performed in a standard soundproof room with a background noise level below 30 dB SPL by the same audiologist.

*Tympanogram* tympanograms were recorded with Titan® device (IMP440—impedance module) from Interacoustics©, Middelfart, Denmark. Tympanometric data were obtained with probe-tone frequencies of 226 Hz. Ear canal pressure was swept in a positive-to-negative direction from + 200 daPa to − 400 daPa. The classification was made according to JERGER’s tympanic pattern classification method [20]. In this study, type A diagram was classified as passing result, and type B and type C diagram as abnormal result.

*DPOAE* DPOAE were recorded with the Titan® device (DPOAE module). The protocol for DPOAE testing consisted of L1/L2 intensity levels of 65/55 dB, F2/F1 ratio of 1.2, and frequency range of 2000–5,000 Hz. DPOAE were considered passing if the SNR exceeded 6 dB in at least 3 of 4 frequency.

*Click ABR* ABR were recorded with the Bio-Logic Navigator PRO system. Stimuli were presented via an insert earphone. Jelly tab® sensors were used to collect responses with a contact impedance of ≤ 5 k Ω and the resistance difference between electrodes is ≤ 2kΩ. The stimulating sound is selected with a duration of 100 μs Click sound, the stimulation rate is 31.1/ times, the alternating wave polarity, the high-pass filter is 100 Hz, the low-pass filter is 1500 Hz, the stacking times are 1000 times, and the window opening time is 16 ms. All evoked potential responses were collected via a single-channel montage where three electrodes were placed on the center of the head (i.e. Cz: non-inverting/ active), the ipsilateral earlobe (i.e. inverting/ reference) and the contralateral earlobe (i.e. ground). Participants were falling sleep in a quiet listening environment during data collection in order to reduce EEG noise. 75 dB nHL Click stimulation sounds were given to both ears twice, respectively. The waveforms were obtained by average superposition and labeled I, III and V waves, and the average incubation period of both ears and the data of I-III, III-V and I-V wave intervals were recorded. The stimulation intensity was decreased until the threshold is obtained. Threshold of wave V greater than 30 dB nHL considers as abnormal ABR.

*PTA* Standard audiometry with pure tone thresholds between 250 and 8000 Hz was conducted using MADSEN Astera2 (type 1066) from Otometrics©, Taastrup, Denmark. Tone average (PTA4) was calculated as the average of air and bone conduction threshold levels at 500, 1000, 2000 and 4000 Hz. PTA4 ≤ 20 dB HL was considered normal hearing. Sensorineural hearing loss (SNHL) is defined as an average air conduction threshold greater than 20 dB HL with an air–bone gap of less than 10 dB. Conductive hearing loss (CHL) is characterized by an average air conduction threshold greater than 20 dB HL, an air–bone gap exceeding 10 dB, and an average bone conduction threshold below 20 dB HL. Mixed hearing loss (MHL) is identified when the average air conduction threshold exceeds 20 dB HL, the air–bone gap is greater than 10 dB, and the average bone conduction threshold is above 20 dB HL. The degree of HL is classified as follows: mild (20- < 35 dB nHL), moderate (35- < 50 dB HL), moderately severe (50- < 65 dB HL), severe (65- < 80 dB HL), and profound (≥ 80 dB HL). Considering the cooperation ability of the children, this test was only administered to those aged older than 5.5 years.

## Retest

All children in the study group were instructed to undergo tympanometry and DPOAE tests one year later, and the results of these two assessments in the first and second times were collected and compared.

### Statistical analysis

The quantitative data conforming to the normal distribution were described as means ± standard deviation. Independent sample t test was conducted in two independent samples comparison. Paired T-tests are used to compare the first and second tests of the same person. One-way analysis of variance and LSD-t test were conducted among multiple groups comparison. Data with non-normal distribution data is described as Median (P25 ~ P75), analyzed by Kruskal–Wallis Test. The qualitative data were described by cases (%), analyzed by Chi-square test or Fisher exact probability test. Significance for *p*-values was set at < 0.05, and two-tailed, if applicable. SPSS27.0 software was used for statistical analysis.

## Results

**Fig. 1:**
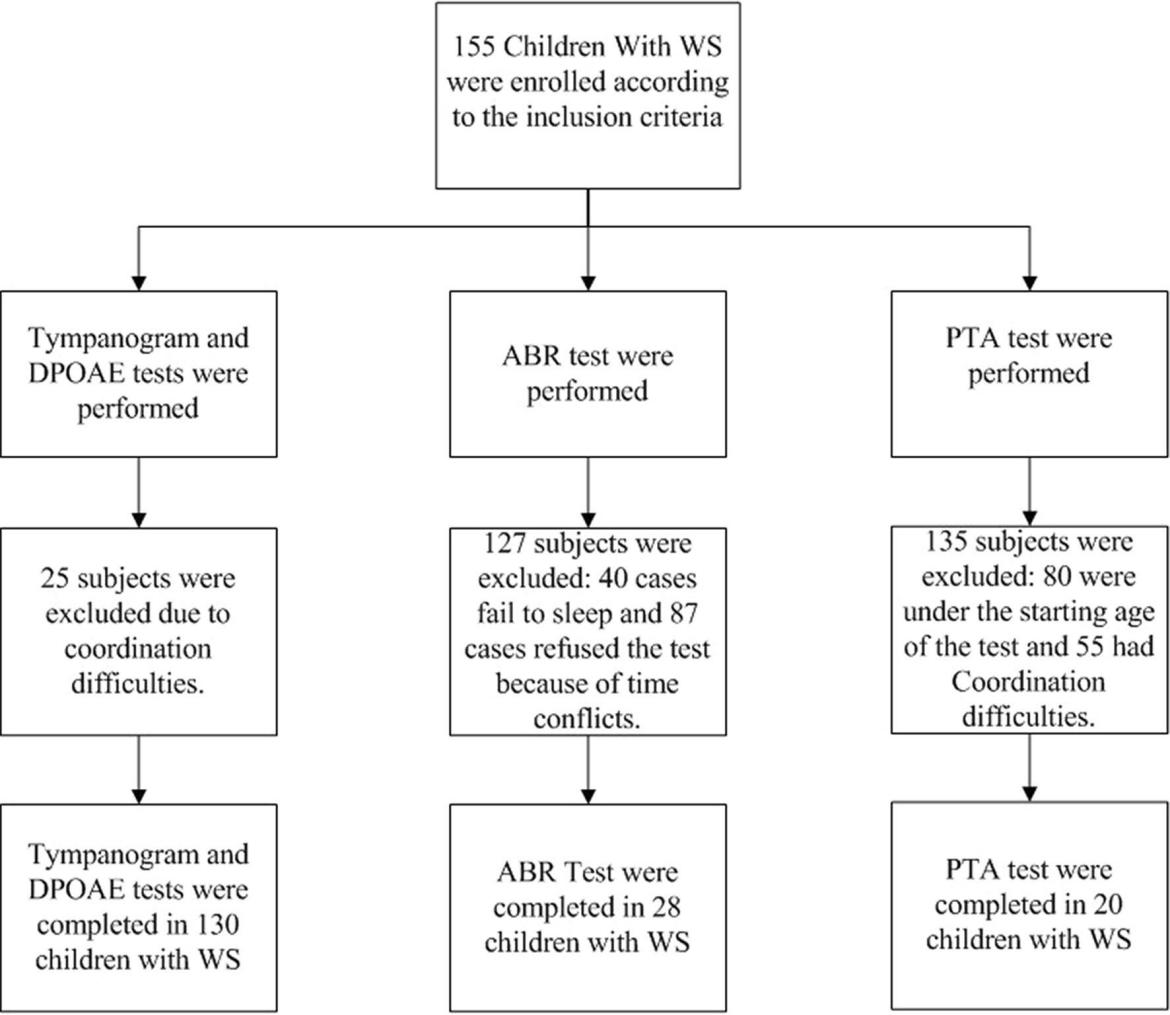
The process of subject enrollment and exclusions in the study group

**Fig. 2:**
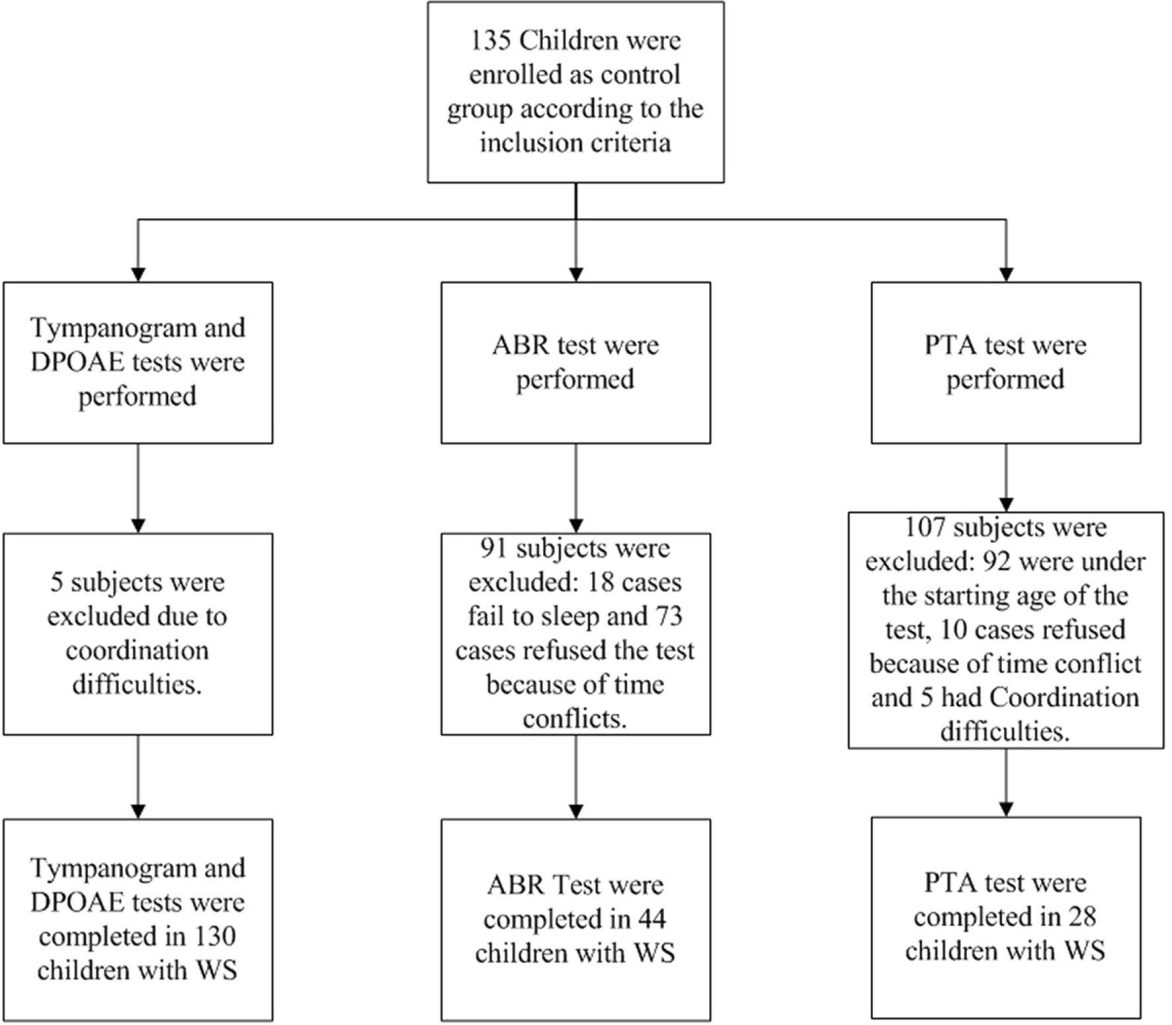
The process of subject enrollment and exclusions in the control group

In tympanogram and DPOAE test, there were 155 cases meeting the inclusion criteria of WS group, and 25 cases with difficulty in cooperation were excluded, 130 cases with 260 ears completed the two tests, the median age is 5.1 years old (ranging from 1.0 to 12.4). 135 children were matched as the control group, and 5 of them with difficulty in cooperation were excluded, 130 children with 260 ears completed the tympanogram and DPOAE test, the median age is 5.3 years old (ranging from 0.8 to 13.1 years). With the ABR test, 40 cases fail to complete the test due to sleep failure, 87 cases refused the test because of time conflicts. 28 cases with a total of 56 ears completed ABR test, the median age is 2.1 years old (ranging from 0.7 to 5.2 years). while in the control group, 18 cases fail to complete the test due to sleep failure. 73 cases refused the test because of time conflicts. 44 cases with a total of 88 ears completed ABR test, the median age is 2.2 years old (ranging from 0.4–5.2 years). The PTA test asks children to raise their hands when they hear a sound, no matter how soft it is. This is very difficult for children with WS because of their cognitive difficulties. Therefore, this study only recruited 20 cases with 40 ears because of above reasons, the median age is 8.5 years old (ranging from 5.9 to 13.7 years). While in the control group, 28 cases with 56 ears completed the PTA test, the median age is 8.4 years old (ranging from 5.9 to 12.15 years). See Table 1 for details. Figures 1 and 2 show the subject enrolment and exclusions for two groups.

**Table 1:** Patient characteristics with different audiology test

	DPOAE or Tympanogram		ABR		PTA	
	WS	Control	WS	Control	WS	Control
N	130	130	28	44	20	28
Median age in years(range)	5.4(1.0–12.4)	5.3(0.8–13.1)	2.1(0.7–5.2)	2.2(0.4–5.2)	8.5(5.9–13.7)	8.4(5.9–12.5)
Age groups(n)						
< 3yrs	24.6% (32)	32.3% (42)	NA	NA	NA	NA
3-5yrs	37.7% (49)	46.2% (60)	NA	NA	NA	NA
> / = 6yrs	37.7% (49)	21.5% (28)	NA	NA	NA	NA
Sex(n)						
Male	58.5% (76)	57.7% (75)	71.4% (20)	68.2% (30)	50% (10)	53.6% (15)
Female	41.5% (54)	42.3% (55)	28.6% (8)	31.8% (14)	50% (10)	46.4% (13)

*NA* Not applicable

### Comparison of tympanogram passing rate between WS and control group

The passing rates of tympanogram in WS and control group were 69.6% (181/260) and 88.8% (231/260) respectively, with statistically significant (χ^2^ = 29.21, *p* < *0.001*). The passing rate was 71.9% (46/64) and 85.7% (72/84) respectively in the < 3 yrs age group, with statistical significance (χ^2^ = 4.30, *p* = 0.038). The passing rate was 67.3% (66/98) and 85.8% (103/120) respectively in the 3–5 yrs age group, with statistical significance (χ^2^ = 10.58, *p* = 0.001). The passing rate was 70.40% (69/98) and 100% (56/56) respectively in the group ≥ 6 yrs old, with statistical significance (χ^2^ = 29.95, *p* < *0.001*).

There was no significant difference in tympanogram passing rate among different age groups in children with WS (*p* > 0.05), while the difference was found in the control group (χ^2^ = 11.92, *p* = 0.002). The passing rate of ≥ 6 yrs age group was significantly higher than that of < 3 yrs age group and 3–5 yrs age group (χ^2^ = 13.00, *p* = 0.002, χ^2^ = 13.85, *p* = 0.001, respectively). No significant difference was found between the other age groups.

### Comparison of DPOAE passing rate between WS and control group

The passing rates of DPOAE in WS and control group were 73.5% (191/260) and 98.5% (256/260) respectively, with statistically significant (χ^2^ = 65.27, *p* < *0.001*). The passing rate was 79.7% (51/64) and 100% (84/84) respectively in the < 3 yrs age group, with statistically significant (χ^2^ = 23.46, *p* < *0.001*). The passing rate was 69.4% (68/98) and 96.7% (116/120) respectively in the 3–5 yrs age group, with statistical significance (χ^2^ = 28.45, *p* < *0.001*). The passing rates were 73.5% (72/98) and 100% (56/56) respectively in the ≥ 6 yrs age group, with statistical significance (χ^2^ = 26.44, *p* < *0.001*). There was no significant difference in DPOAE passing rate among different age groups, neither in WS nor control group (*p* > 0.05).

### Comparison of DPOAE passing rate between WS and control group in cases with passing tympanogram

In this part, 79 ears in WS group and 29 ears in control group failed passing the tympanogram were excluded. The DPOAE passing rate was 89.0% (161/183) and 99.6% (227/231) respectively after exclusion, with statistically significant (χ^2^ = 21.19, *p* < *0.001*). The passing rate was 89.1% (41/46) and 100% (70/70) respectively in the < 3 yrs age group, with statistical significance (χ^2^ = 9.59, *p* = 0.009). The passing rate was 89.4% (59/66) and 99% (101/102) respectively in 3–5 yrs age group, with statistical significance (χ^2^ = 6.20, *p* = 0.013). The passing rate was 88.4% (61/69) and 100% (56/56), respectively in the group ≥ 6 yrs old, with statistical significance (χ^2^ = 9.95, *p* = 0.008). There was no significant difference in DPOAE passing rate among different age groups, neither in WS nor control group (*p* > 0.05).

### Comparison of DPOAE SNR between WS and control groups in cases with passing tympanogram and DPOAE

In this part, cases didn’t pass either tympanogram or DPOAE were excluded, resulting in 161 ears in the WS group and 226 ears in the control group. It was found that the SNR of 2000, 3000, 4000, 5000 Hz were statistically lower in WS group (*p* < 0.05), and in all age groups. There was no difference in WS group of SNR among different age groups, while the SNR of the control group over 3 yrs old was higher than that of the group under 3 yrs old. See Table 2 for the details.

**Table 2:** Comparison of SNR between WS and control group (dB)

	2000 Hz				3000 Hz				4000 Hz				5000 Hz			
	WS	Control	Z	*p*	WS	Control	Z	*p*	WS	Control	Z	*p*	WS	Control	Z	*p*
< 3Y	11.0(9,16.4)	14.5(11.1,19.5)	− 2.53	**0.011**	13.6(8.9,21.7)	16.7(12.4,22.9)	− 1.948	**0.045**	14.6(11.5,21.2)	20.5(15.0,25.2)	− 3.00	**0.003**	17.2(12.4,21.8)	21.4(14.4,27.9)	− 2.45	**0.014**
3− 5Y	9.7(8.5,12.9)	18.2(12.6,21.9)	− 6.57	** < 0.001**	12.2(9.1,16.4)	19.5(16.8,23.3)	− 6.228	** < 0.001**	15.0(10.4,21.8)	24.6(15.7,28.6)	− 4.70	** < 0.001**	19.5(13.7,25.5)	25.8(19.6,31.1)	− 4.29	** < 0.001**
≥ 6Y	10.7(7.8,15.8)	18.2(13.7,22.4)	− 4.98	** < 0.001**	13.4(9,18.5)	20.4(18.2,24.3)	− 5.186	** < 0.001**	16.5(12.7,23.9)	26.3(18.2,28.9)	− 4.04	** < 0.001**	19.7(12.7,28.3)	28.0(19.18,30.9)	− 3.13	**0.002**
Total	10.6(8.5,14.7)	17.0(12.2,21.7)	− 8.42	** < 0.001**	13.0(9.0,18.5)	19.3(15.3,23.4)	− 7.631	** < 0.001**	15.0(11.7,22.7)	23.4(16.5,28.4)	− 6.69	** < 0.001**	18.8(13.0,25.0)	25.0(18.2,30.8)	− 5.66	** < 0.001**
H	1.82	8.74			1.80	9.80			1.88	8.93			3.07	12.13		
P	0.401	**0.013**			0.405	**0.007**			0.39	**0.011**			0.215	**0.002**		

The data are expressed as the Median (P25 ~ P75)

Statistically significant differences are highlighted in bold

### Change in the passing rate of retest

Tympanogram and DPOAE reexamination were performed in 25 WS patients in the following 1 years, with a total of 50 ears. The first examination age was 4.6 (1.0–7.9) yrs old, and the reexamination age was 5.8 (2.3–9.8) yrs old.

In the tympanogram test, 15 ears were both abnormal in the two visits, 21 ears were both passing, 8 ears changed from passing to abnormal, and 6 ears changed from abnormal to passing. The passing rate was 58% (29/50) and 54% (27/50) respectively, with no difference (*p* > 0.05). In DPOAE test, 11 ears were both abnormal in the two visits, 29 ears were both passing. 7 ears changed from passing to abnormal, and 3 ears changed from abnormal to passing. The passing rate was 74% (36/50) and 64% (32/50), respectively with no difference (*p* > 0.05).

### Comparison of ABR results between WS and control group

28.6% (16/56) and 4.5% (4/88) was found abnormal ABR in WS and control group respectively, with statistical difference (χ^2^ = 14.57, *p* < *0.001*). The threshold of ABR is 17.5(10,38.8) dB nHL in the WS group, significantly higher than that in the control group [15(10,20) dB nHL, Z = −3.22, *p* = 0.001]. Since ABR results were easily affected by the function of the middle ear and cochlea, data with either tympanogram refer, DPOAE refer, ABR threshold greater than 30 dB nHL were excluded when analyzing ABR latency results. The number of ears were 32 and 84 in WS and control group after exclusions, respectively. It was found that the latency of wave I and III and the interpeak latency I-III in WS group were significantly longer than that in control group (*p* < *0.05*), and the interpeak latency III-V was significantly shorter than that in control group (*p* < *0.05*). See Table 3 for the details.

**Table 3:** The comparison in ABR latency and interpeak latency between WS group and the control group (ms)

	WS group	Control group	T	*p*
N	33	84		
Wave I	1.7 ± 0.1	1.6 ± 0.1	5.21	** < 0.001**
Wave III	4.1 ± 0.3	3.9 ± 0.2	4.64	** < 0.001**
Wave V	5.9 ± 0.3	5.8 ± 0.3	1.35	0.179
Interpeak latency I-III	2.5 ± 0.3	2.3 ± 0.2	2.43	**0.017**
Interpeak latency III-V	1.8 ± 0.2	1.9 ± 0.2	− 4.31	** < 0.001**
Interpeak latency I-V	4.2 ± 0.3	4.3 ± 0.3	− 0.67	0.504

The data are expressed as the Mean ± SD

Statistically significant differences are highlighted in bold

### Comparison of PTA results between WS and control group

50% (20/40) in WS group were found with HL, with CHL in 60% (12/20), SNHL in 20% (4/20), MHL in 20% (4/20). And among these people, 65% (13/20) of HL were mild, 25% (5/20) were moderate, 5% (1/20) was moderate-severe, 5% (1/20) was severe. 3.6% (2/56) in the control group were found HL and all were mild. The incidence rate of HL was statistical difference when compare with WS group (χ^2^ = 25.91, *p* < *0.001*). The threshold of 250–8000 Hz in WS group were significantly higher than that in control group (*p* < *0.05*), either in air or bone conduction test. The mean air–bone gap in WS group were significantly higher than that in control group (*p* < *0.05*). The comparison of threshold values of 250–8000 Hz between the two groups is shown in the Table 4.

**Table 4:** Comparison of PTA between WS and control group (dB HL)

	Frequency (Hz)	WS group	Control group	Z	*p*
Air-conduction	250	25 (20,30)	20 (15,20)	− 5.29	** < 0.001**
	500	25 (20,25)	15 (15,20)	− 5.17	** < 0.001**
	1000	20 (15,25)	15 (10,20)	− 5.34	** < 0.001**
	2000	20 (15,25)	15 (10,15)	− 4.57	** < 0.001**
	4000	20 (15,32.5)	15 (10,15)	− 5.30	** < 0.001**
	8000	20 (15,25)	10 (10,15)	− 6.09	** < 0.001**
	Mean threshold	20.63 (15,25)	14.38 (11.6,17.2)	− 5.57	** < 0.001**
Bone-conduction	250	10 (10,15)	10 (10,13.8)	− 2.77	**0.006**
	500	15 (10,15)	10 (5,13.8)	− 3.85	** < 0.001**
	1000	12.5 (10,20)	10 (5,10)	− 4.36	** < 0.001**
	2000	10 (6.25,20)	10 (5,10)	− 2.94	**0.003**
	4000	10 (10,20)	5 (5,10)	− 5.26	** < 0.001**
	8000	10 (10,20)	5 (5,10)	− 5.95	** < 0.001**
	mean threshold	11.25 (8.75,22.19)	8.75 (6.3,11.3)	− 4.53	** < 0.001**
	mean air–bone gap	8.75 (2.5,11.25)	5 (3.8,7.2)	− 2.38	**0.018**

The data are expressed as the Median (P25 ~ P75)

Statistically significant differences are highlighted in bold

## Discussion

This study found that children with WS across various age groups had a lower tympanogram passing rate compared to the control group, indicating a high prevalence of middle ear dysfunction that persists from early childhood (before 3 years old) and does not improve with age. In contrast, the control group showed a higher tympanogram passing rate, which increased after 6 years old. Otitis media, a common complication in WS, is the primary cause of tympanogram abnormalities, with recurrent otitis media reported in up to 50% of cases [13]. While some studies, such as Oliveira’s, found normal tympanic membrane function in WS, but their subjects were older (average age > 12 years) [21]. Klein’s questionnaire-based study reported 61% of WS individuals had recurrent otitis media or tympanic catheterization, aligning with our findings, though their data relied on parental recall and included a wide age range (1–28 years) [22]. In this study, the passing rate of tympanogram of the control group increased after the age of 6 years. The lower passing rate in younger children may be attributed to a flat eustachian tube, improper feeding posture, or respiratory infections, which can facilitate the spread of nasopharyngeal secretions to the middle ear, causing otitis media. With age, eustachian tube maturation and improved tilt angle significantly reduce otitis media incidence. Thus, control group results reflect feeding behavior changes and ear structure maturation, while performance differences in the WS group may be linked to gene deletion. Elastin, encoded by the ELN gene, is a crucial component of elastic fibers in connective tissues and essential for normal middle ear development and function. ELN gene deletion in WS reduces eustachian tube tension, impairing its opening during palatine tensor muscle contraction and hindering middle ear secretion drainage, predisposing to otitis media. Increased stiffness of the tympanic membrane and stapes tendons further disrupts sound transmission and middle ear function. The consistent passing rates in the first and second examinations suggest that middle ear dysfunction in this population may be persistent or recurrent.

This study demonstrated a significantly reduced DPOAE passing rate in WS across all age groups. Excluding abnormal tympanogram data increased the passing rate from 73.5% to 89%, yet it remained consistently lower than in controls. Even without overt hearing abnormalities (e.g., normal tympanogram and DPOAE results), subclinical cochlear dysfunction (e.g., reduced SNR) may persist. These findings suggest that both overt and subclinical cochlear dysfunction are prevalent in this population, often emerging before3 years old. Alessia [23] reported reduced amplitudes and prolonged latencies of transient evoked otoacoustic emissions (TEOAE) and narrowband TEOAE in WS compared to controls, indicating subclinical cochlear dysfunction even in individuals with normal hearing, consistent with our findings. As previously noted, elastin deficiency increases basement membrane stiffness, thickens and narrows blood vessel walls [24]. Increased stiffness reduces shear motion of the basement and operculum membranes during sound stimulation, impairing mechanoelectrical transduction and hair cell signaling. Thickened and narrowed vessels cause cochlear hypoperfusion, leading to hypoxia and hair cell death, contributing to cochlear dysfunction. Thus, early DPOAE monitoring is recommended to detect mild hearing dysfunction in order to avoid exacerbating the language or cognitive impairment related to WS. Unlike previous studies, we did not observe reduced high-frequency SNR in children with WS, potentially due to age differences. Alessia’s subjects (7–23 years) exhibited age-related high-frequency hearing decline, but the small sample size (n = 13) limits generalizability. Our study focuses on a younger population, and extended follow-up will further elucidate hearing changes over time.

The elevated ABR threshold in WS indicates hearing abnormalities in this group. After excluding tympanogram or DPOAE abnormalities, WS showed prolonged latencies for waves I and III and interpeak latency I-III compared to controls, but a shorter interpeak latency III-V. While prolonged ABR latencies are common in neurodevelopmental disorders, a shortened interpeak latency III-V is atypical [25, 26]. Barozzi tested ABR in 14 individuals with WS (aged 9–30 years) and found no abnormalities in threshold, latency, or interpeak latency [27]. Gothelf reported prolonged latencies for waves I, III, and V in WS but did not observe a shortened interpeak latency [28]. These latency abnormalities may have been influenced by middle ear dysfunction, which affects auditory processing. However, Gothelf’s study included older participants (6–26 years) and lacked bone conduction ABR or tympanogram data, making it difficult to identify the specific auditory pathway responsible for the prolonged latency. Nascimento found increased latency of wave III in adults with WS [29], but their study did not exclude subjects with HL, potentially confounding the ABR results. ABR waves I, III, and V represent the action potentials of auditory nerve fibers, cochlear nucleus neurons, and the central nuclear mass of the inferior colliculus, respectively, reflecting the speed of peripheral-to-central sound transmission [30]. As auditory nerve fibers develop, synaptic structure and function improve, leading to a gradual shortening of ABR latency until stabilization [31]. Previous studies report prolonged interpeak latencies III-V and I-V in children with general developmental delay (GDD) under 3 years [32]. Cognitive developmental delay is related to the immature state of neural connections or cortical development in the brain, while overall immaturity may show a longer latency or interpeak latency in the auditory nerve portion. GDD, common in WS, may explain the prolonged latency of waves I and III and interpeak latency I-III. However, this study identified a shortened interpeak latency III-V in WS children. The FZD9 gene, frequently deleted in WS, is expressed in the cochlear spiral ganglion and regulates neurite regeneration in mature spiral ganglion neurons [33]. This may influence auditory nerve impulse conduction, though the mechanism remains unclear. Auditory hypersensitivity, observed in 77–100% of WS [34], has been linked to sensory neuron degeneration. Degeneration of some neurons may cause overcompensation in remaining neurons, leading to "over-adaptation" [35], potentially explaining the shortened interpeak latency III-V. It was speculated that children with WS may have an overactive central auditory nervous system or a loss of inhibition, especially the electrical activity of the central nucleus of the hypothalamu. These factors shorten the interpeak latency in the central segment of ABR, leading to abnormally reduced interpeak latency III-V. Previous studies suggest that auditory hypersensitivity diminishes with age, potentially explaining the absence of shortened ABR interpeak latency in older patients. Thus, the shortened interpeak latency III-V may serve as a biomarker in children with WS.

The study revealed that HL is common in children with WS, arising not only from middle ear dysfunction but also from inner ear abnormalities and/or retrocochlear neuropathy, primarily manifesting as mild impairment. This explains why there is such a high incidence of HL but so few parents report hearing problems. Deletion of the ELN gene mentioned above leads to abnormal functioning of the middle and inner ears, which may result in CHL and SNHL, respectively. The LIMK1 gene, located within the WS deletion region, encodes a serine/threonine kinase regulating actin reorganization. Reduced expression impairs actin dynamics. Matsumoto [36] demonstrated its expression in cochlear outer hair cells (OHCs), where LIMK1-mediated cofilin phosphorylation inhibits actin depolymerization, enhancing OHC electromotility and cell length. LIMK1 deficiency reduces electromotility and shortens OHCs, potentially weakening basilar membrane vibration, diminishing cochlear amplifier gain, and contributing to SNHL. Another commonly deleted gene, GTF2IRD1, encodes Transcription Factor II-I, a multifunctional transcription factor influencing craniofacial morphology and neurocognitive features in WS. Its role in auditory function remains under investigation. Canales [37] demonstrated that the GTF2IRD1 gene is expressed in various cochlear structures in mice, including OHCs, supporting cells, the stria vascularis, spiral limbus, and interdental cells. GTF2IRD1 knockout mice exhibited normal ABR latencies but elevated thresholds, decreased SNR of DPOAE, and flattened input/output curves, indicating cochlear dysfunction. The observed SNHL was mild, with no significant cochlear structural damage, suggesting pathology primarily in OHCs or other GTF2IRD1-expressing cells. For example, interdental cell defects may reduce CEACAM16 production [38], while stria vascularis or supporting cell defects may disrupt potassium ion recycling [39], impairing endocochlear potential and cochlear amplification, ultimately causing SNHL. When the aforementioned mechanisms of CHL and SNHL occur simultaneously, MHL ensues. Barozzi monitored PTA in WS over 5–10 years, finding no significant change in HL incidence within five years, though high-frequency hearing abnormalities increased. After ten years, both average hearing thresholds and high-frequency hearing declined [40]. Another study reported significantly higher high-frequency thresholds in individuals over 15 years compared to those under 15 [27]. The study did not reveal a significant trend of high-frequency HL, potentially due to the subjects’ age. However, due to the small number of cases, it is still necessary to further expand the sample size to obtain more valuable conclusions. A substantial proportion of HL cases were associated with peripheral auditory system disorders, mainly middle ear dysfunction (e.g., otitis media), which is generally more treatable than inner ear or retrocochlear issues. Nonetheless, extended observation is required to monitor long-term changes in these children’s PTA results.

## Conclusions

This study employs a combined subjective and objective approach to evaluate auditory function in children with WS across age groups. The results indicate that children with WS often experience abnormal middle ear and inner ear functions, leading to HL, which can begin as early as 3 years old. A significant portion of HL in these children is attributed to middle ear problems, which are potentially treatable. Subclinical HL also exists in WS with normal hearing, which was certificated by lower SNR. Increased PTA and ABR threshold also suggest that HL in this group can’t be ignored. In terms of auditory nerve response, it was also found that the maturation speed of each wave was slow, but the conduction speed of the near central segment was abnormally fast, indicating that children with WS had abnormal auditory nerve processing problems. Hearing performance in WS may be related to recurrent otitis media, auditory hypersensitivity and cochlear active mechanism dysfunction caused by genetic problems. For early diagnosis and early intervention, a long-term hearing follow-up program should be established for children with WS to regularly monitor the functions of the middle ear, inner ear, and auditory nerve. A long-term hearing follow-up program, including annual audiological assessments (tympanometry, DPOAE, PTA, ABR), is recommended to detect HL onset and monitor progression. Even in cases of normal hearing, cochlear function should be evaluated and tracked over time.

## Data Availability

All relevant data generated or analyzed during this study are included in this published article. If further datasets are requested, these are available on request from the corresponding author.
